# Supplementary material for: Role for formin-like 1-dependent acto-myosin assembly in lipid droplet dynamics and lipid storage
Source: Nat Commun. 2017 Mar 31;8:14858. doi: 10.1038/ncomms14858 (PMC5380971; doi:10.1038/ncomms14858)
Supplement: Supplementary Information — Supplementary Figures and Supplementary Table [file ncomms14858-s1.pdf]

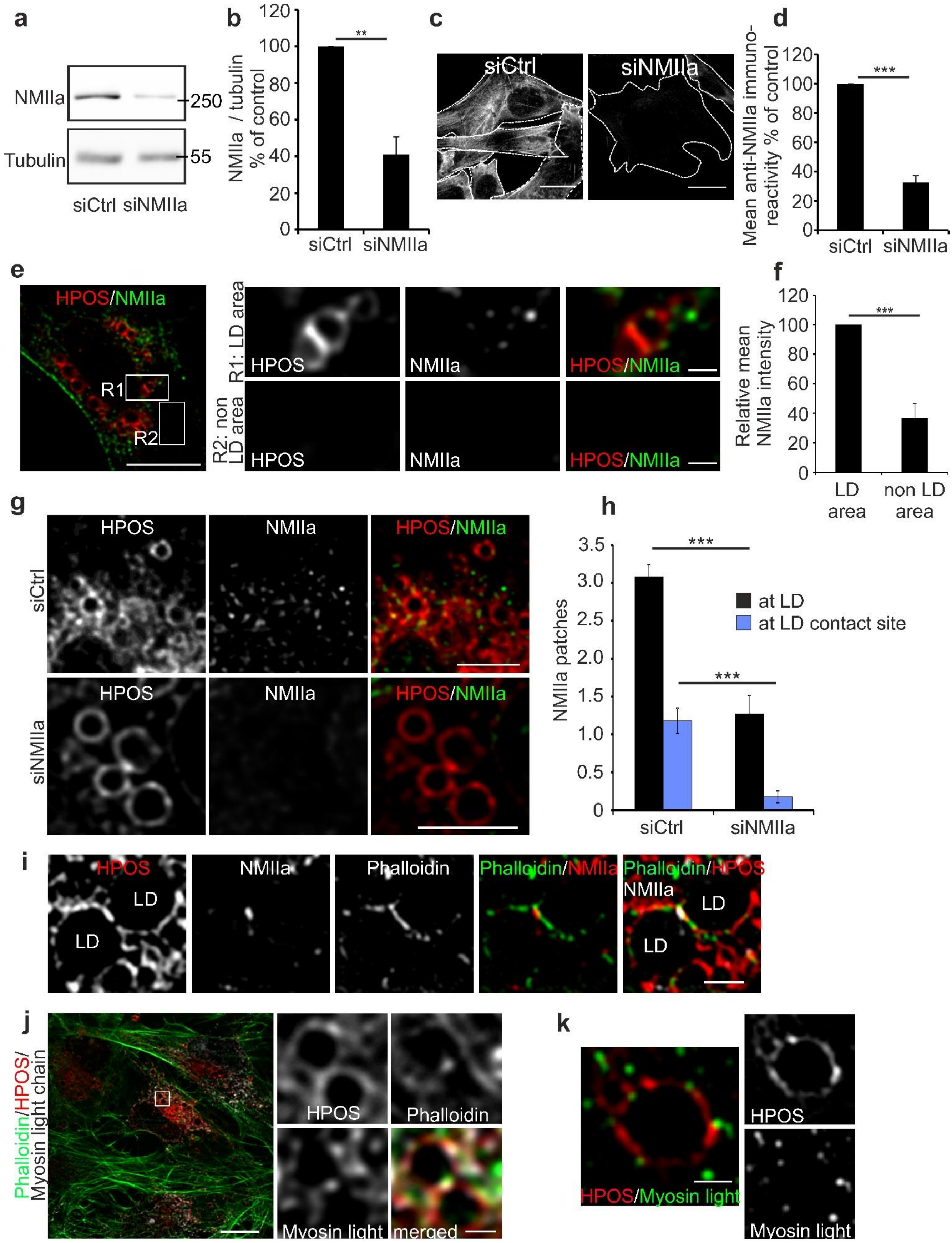
a
NMIIa
Tubulin
250
55
siCtrl siNMIIa
b
NMIIa / tubulin % of control
**
siCtrl siNMIIa
c
siCtrl
siNMIIa
d
Mean anti-NMIIa immuno-reactivity % of control
***
siCtrl siNMIIa
e
HPOS/NMIIa
R1
R2
R1: LD area
R2: non LD area
HPOS
NMIIa
HPOS/NMIIa
f
Relative mean NMIIa intensity
***
LD area
non LD area
g
HPOS
NMIIa
HPOS/NMIIa
siCtrl
siNMIIa
h
NMIIa patches
at LD
at LD contact site
***
siCtrl siNMIIa
i
HPOS
NMIIa
Phalloidin
Phalloidin/NMIIa
Phalloidin/HPOS
NMIIa
LD
j
Phalloidin/HPOS/ Myosin light chain
HPOS
Phalloidin
Myosin light
merged
k
HPOS/Myosin light
HPOS
Myosin light

Supplementary Figure 1. Transient reduction of NMIIa protein expression. **a**, U2OS cells were treated with siCtrl or siNMIIa and cell extracts were subjected to western blot analysis by Odyssey detection using anti-NMIIa and anti-tubulin antibodies. **b**, NMIIa protein abundance was quantified relative to tubulin using Odyssey quantification tools. ± SEM, n = 5 samples from 4 independent experiments **c**, Stable U2OS Cherry-HPOS (not shown) cells were treated with siCtrl or siNMIIa and were loaded with 400 µM oleic acid overnight. Cells were fixed and immunostained for NMIIa. Scale bar 20 µm. **d**, Quantification of mean cellular NMIIa intensity of NMIIa silenced cells relative to control cells. Cells were treated as in c. ± SEM n = 13 images from 2 independent experiments. **e,** Stable U2OS Cherry-HPOS cells were treated with 400 µM oleic acid, saponin permeabilized, fixed and immunostained for NMIIa. Same cell as in Fig. 2a is displayed, with a LD area R1 and non-LD area R2 magnified. Scale bar 10 µm and 1 µm (inset). **f**, Quantification of mean NMIIa intensities in LD areas or areas containing no LDs and no prominent stress fibers. n = 10 cells from 2 independent experiments. **g**, Stable U2OS Cherry-HPOS cells were treated with siControl or siNMIIa for 48 h and with 400 µM oleic acid overnight. Cells were permeabilized with saponin, fixed and immunostained for NMIIa. Scale bar 5 µm. **h**, Quantification of NMIIa patches at LDs from images acquired as in (**g**), n = 50 LDs for siCtrl and 40 LDs for siNMIIa. NMIIa labeling at LDs was specific as evidenced by reduced labeling upon NMIIa silencing. **i**, 3D-SIM of U2OS Cherry-HPOS cells permeabilized with saponin, fixed and immunostained for NMIIa and with Alexa488-phalloidin, scale bar 1 µm. **j**, **k**, Myosin light chain localization at LDs. Stable U2OS Cherry-HPOS cells were treated with 400 µM oleic acid overnight, permeabilized with saponin, fixed and immunostained for myosin light chain (and with Alexa 488 phalloidin, **j**). Images were acquired by confocal microscopy (**j**) or 3D-SIM (**k**). Scale bar 10 µm and 1 µm (inset) in (**j**) and 0.5 µm in (**k**). Student's t-test, ** $p < 0.01$, *** $p < 0.001$.

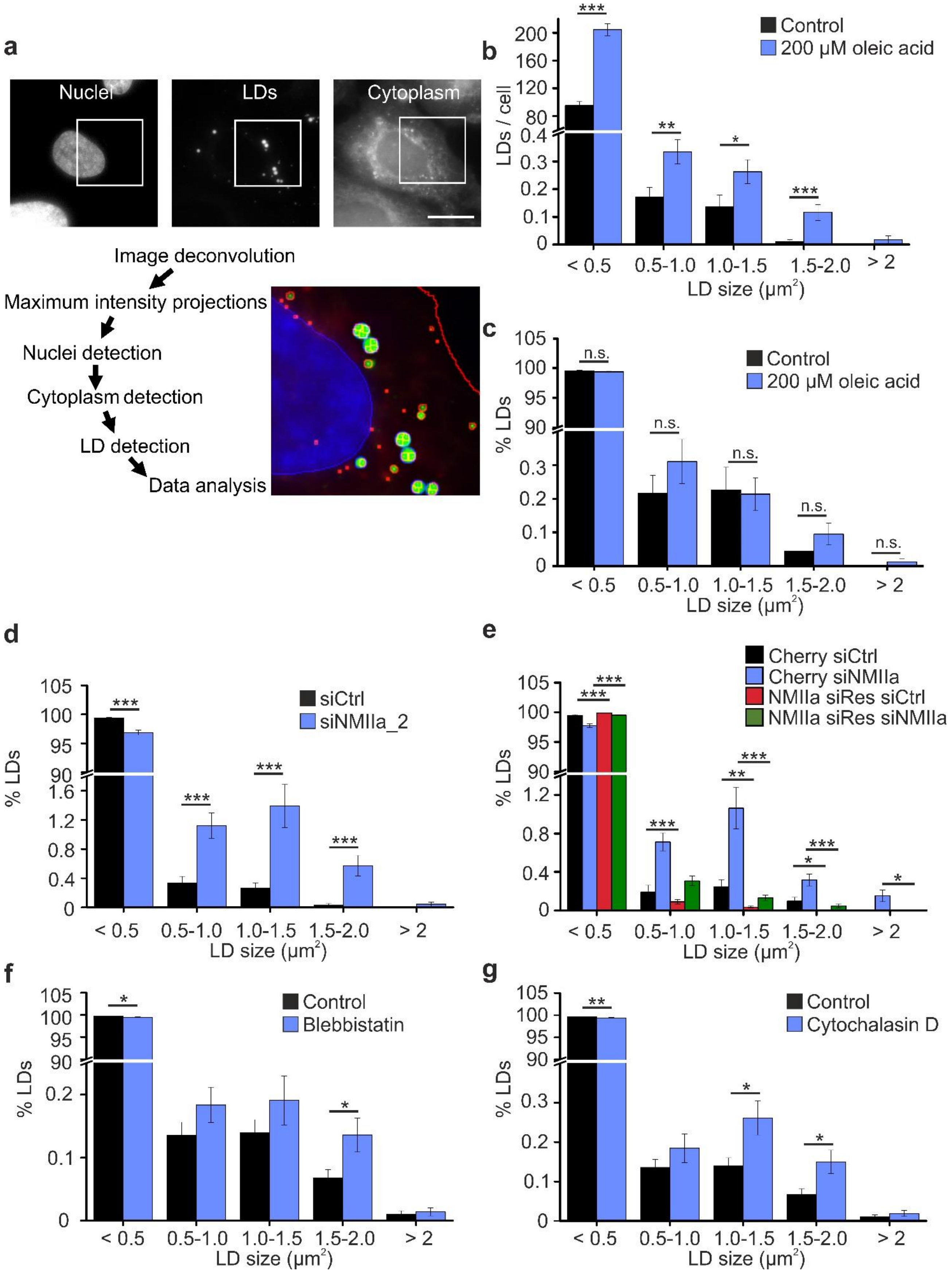
a
Nuclei
LDs
Cytoplasm
Image deconvolution
Maximum intensity projections
Nuclei detection
Cytoplasm detection
LD detection
Data analysis
b
Control
200 μM oleic acid
LDs / cell
LD size (μm²)
< 0.5
0.5-1.0
1.0-1.5
1.5-2.0
> 2
c
Control
200 μM oleic acid
% LDs
n.s.
LD size (μm²)
d
siCtrl
siNMIIa_2
% LDs
LD size (μm²)
e
Cherry siCtrl
Cherry siNMIIa
NMIIa siRes siCtrl
NMIIa siRes siNMIIa
% LDs
LD size (μm²)
f
Control
Blebbistatin
% LDs
LD size (μm²)
g
Control
Cytochalasin D
% LDs
LD size (μm²)

Supplementary Figure 2. a, Schematic outline of the steps involved in automated LD analysis. Z-stack images of nuclei, LDs and cytoplasm were acquired using a Nikon Eclipse Ti-E inverted microscope with at least 10 image stacks per treatment and experiment. Images were automatically deconvolved and maximum projections were made. The resulting images were analyzed using CellProfiler for cell and LD detection. Small LDs are encircled in red and large LDs in blue. Please note that misclassification of large LDs as several small LDs was corrected during data analysis. The resulting image data was further processed using MatLab and Excel, graphs were generated using OriginPro 8.6. scale = 20 µm. **b,** Automated LD quantification of U2OS cells cultured in control medium or treated with 200 µm oleic acid for 7 h. ± SEM, n = 199 cells for control medium and 244 cells for oleic acid treatment. (Note, same data as in Fig. 1 b, d; siCtrl). **c**, LD quantification presented as % cellular LDs for individual size ranges (same data as in b). **d,** Automated LD quantification of U2OS cells treated with siCtrl or an independent siRNA targeting NMIIa (siNMIIa_2). ± SEM, n = 282 cells for siCtrl and 232 for siNMIIa_2 from 3 independent experiments. **e,** LD quantification of siCtrl and siNMIIa treated stable U2OS Cherry and U2OS Cherry-NMIIa siRes cells, loaded with 200 µM oleic acid for 8 h. ± SEM, n = 236 for Cherry siCtrl, 172 cells for Cherry siNMIIa, 215 cells for Cherry-NMIIa siRes siCtrl and 163 cells for Cherry-NMIIa siRes siNMIIa from 3 independent experiments. **f**, LD quantification of U2OS cells treated with 400 µM oleic acid for 8 h with or without 30 µM blebbistatin during the last hour of incubation. ± SEM, n = 480 cells for control and 461 cells for blebbistatin from 3 independent experiments. **g**, Experiment was performed as in **f**, but using 2 µM cytochalasin D instead of blebbistatin, control cells are the same as in (**f**), ± SEM, n = 480 cells for control and 391 cells for cytochalasin D treatment from 3 independent experiments. Student's t-test, * $p < 0.05$, ** $p < 0.01$, *** $p < 0.001$.

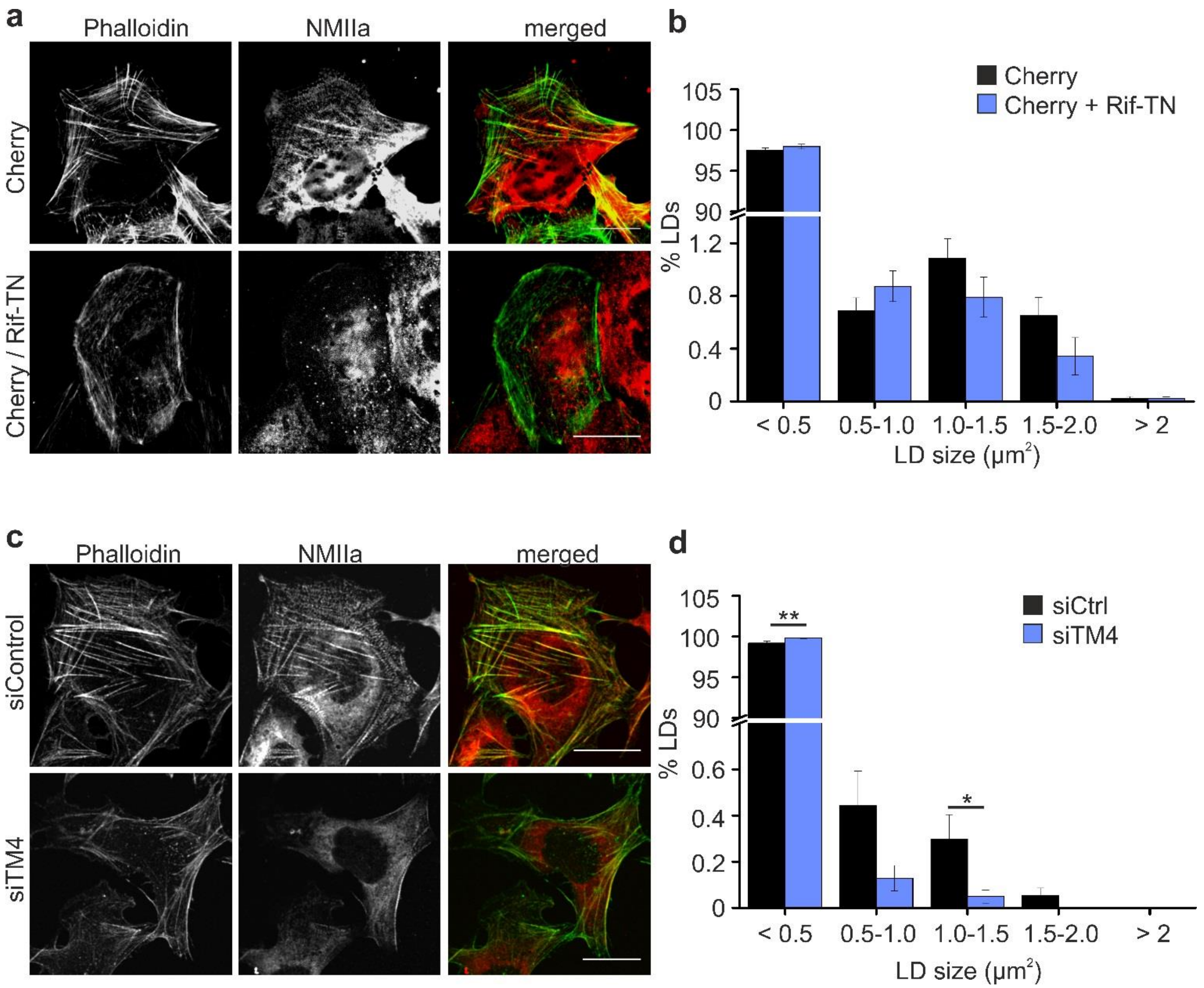

Supplementary Figure 3. Disruption of NMIIa positive stress fibers via transient expression of dominant negative RIF (RIF-TN) or TM4 silencing does not evoke the formation of large LDs. **a**, Transient Rif-TN expression results in the disruption of NMIIa positive stress fibers. U2OS cells were transfected with Cherry or Cherry plus Rif-TN expression plasmids and incubated for 24 h in complete medium. Cells were fixed and immunostained with anti-NMIIa , anti-rabbit Alexa 647 and Alexa 488 phalloidin. Transfected cells are indicated with white lines, scale bar 20 µm. **b**, Automatic LD quantification of U2OS cells transiently expressing Cherry or Cherry plus Rif-TN for 24 h including an overnight load with 200 µM oleic acid. ± SEM, n = 74 cells for both Cherry or Cherry plus Rif-TN from 2 independent experiments. (Note, plasmid transfection reagent tends to increase LD size.) **c,** Depletion of TM4 reduces NMIIa positive stress fibers. U2OS Cherry-HPOS cells were treated with siCtrl or siTM4 for 72 h and immunostained with anti-NMIIa , anti-rabbit Alexa 647 and Alexa 488 phalloidin. Scale bar 20 µm. **d,** Automated LD quantification of U2OS cells expressing siCtrl and siTM4 for 72 h. ± SEM, n = 168 cells for siCtrl and 138 for siTM4 from 2 independent experiments. Significant differences are indicated with asterisks, Student's t-test, * $p < 0.05$, ** $p < 0.01$.

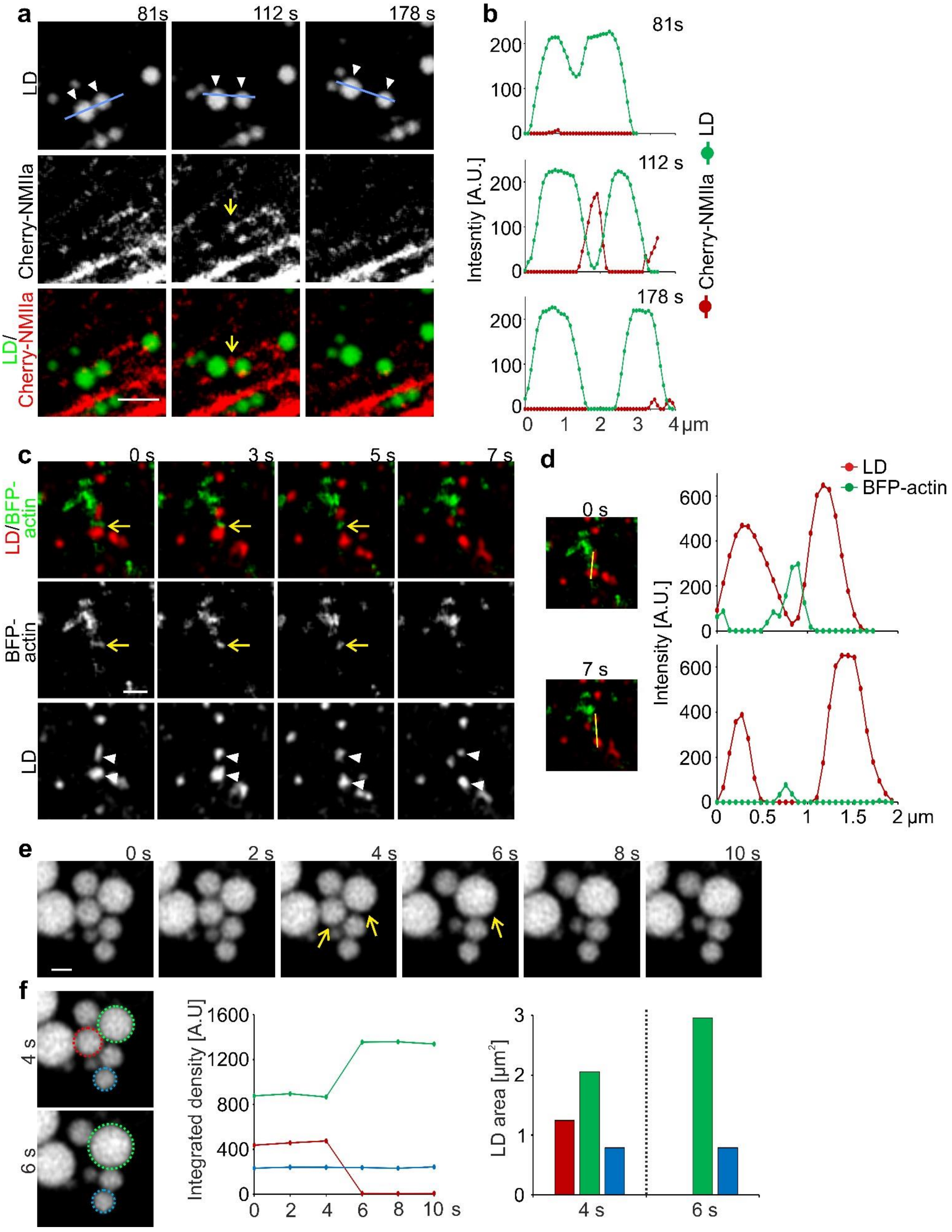

a
81s
112 s
178 s
LD
Cherry-NMIIa
LD/ Cherry-NMIIa
b
81s
112 s
178 s
Intesntiy [A.U.]
LD
Cherry-NMIIa
0 1 2 3 4 µm
c
0 s
3 s
5 s
7 s
LD/BFP-actin
BFP-actin
LD
d
0 s
7 s
LD
BFP-actin
Intensity [A.U.]
0 0.5 1 1.5 2 µm
e
0 s
2 s
4 s
6 s
8 s
10 s
f
4 s
6 s
Integrated density [A.U]
0 2 4 6 8 10 s
LD area [µm2]
4 s
6 s

Supplementary Figure 4. NMIIa and actin transiently localize to LD dissociation sites. **a**, Live cell microscopy of U2OS cells stably expressing Cherry-NMIIa treated with 200 µM oleic acid overnight. Live cell imaging was performed in medium without oleic acid and images were acquired at 1.72 s intervals. Arrows indicate transient Cherry-NMIIa accumulation between dissociating LDs (arrowheads), scale bar 2.5 µm. **b**, Intensity line profiles of the indicated areas in (**a**). **c**, Live cell Airyscan microscopy of U2OS cells transfected with BFP-actin for 24 h and loaded with 400 µM oleic acid overnight. Arrows indicate BFP-actin between dissociating LDs (indicated with arrowheads). Frame rate 1 s, scale bar 1 µm. **d**, Intensity line profiles to visualize transient BFP-actin accumulation between LDs in the images indicated. **e**, Fusion of associated LDs. U2OS cells were treated with 400 µM oleic acid overnight and imaged by live cell Airyscan microscopy. Images were acquired every 2 s for 5 min. Scale bar 1 µm. Arrows indicate fusing LDs. **f,** Quantification of integrated densities and LD areas of fusing LDs. LD marked with red fuses with LD marked with green. Blue delineates a non-fusing control LD. This figure is accompanied by Supplementary Movies 2, 3)

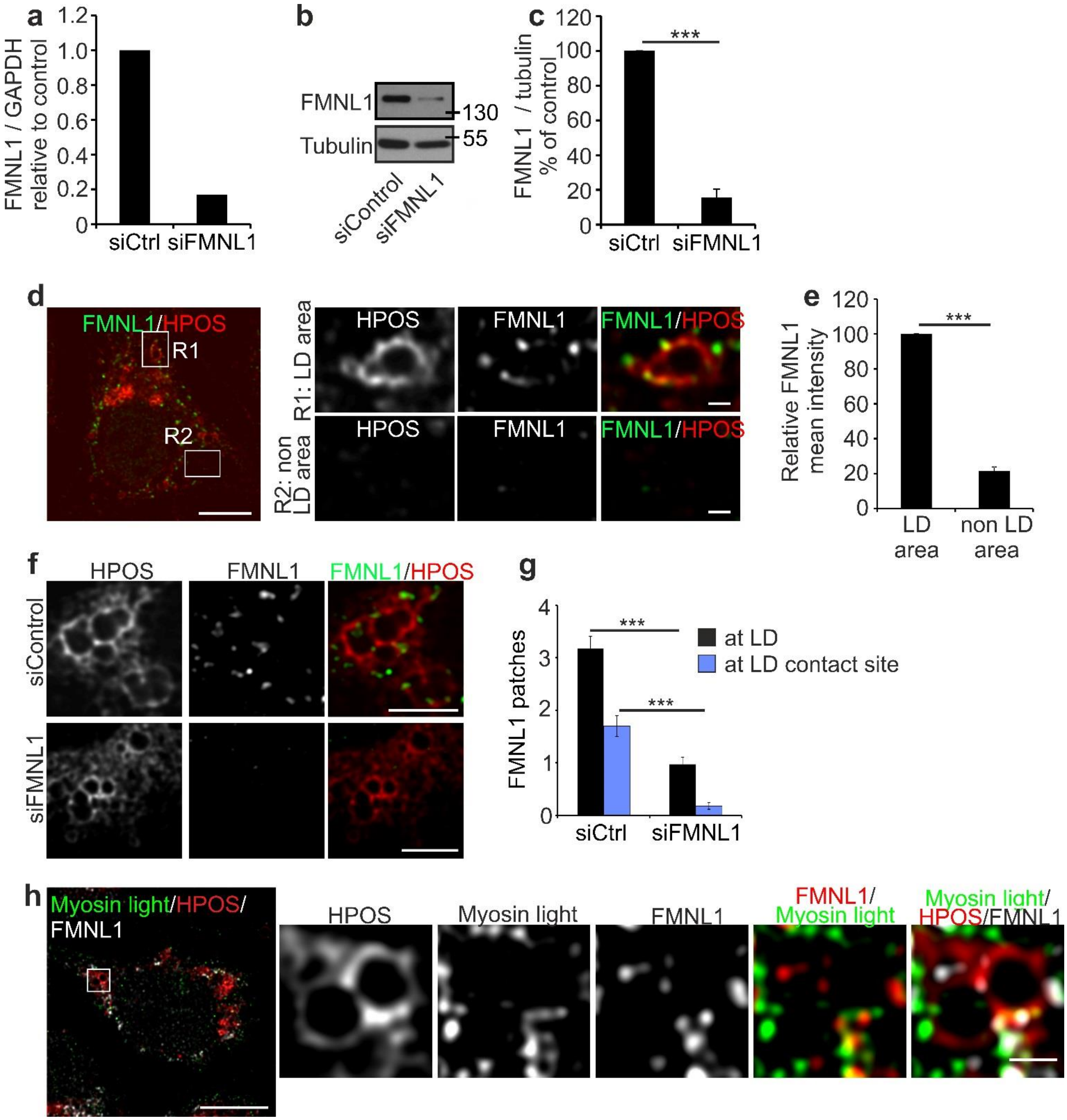

Supplementary Figure 5. a, U2OS cells were treated with control and FMNL1 siRNAs for 24 h and FMNL1 mRNA expression was analysed by quantitative PCR and normalized to GAPDH expression. **b**, U2OS cells were treated with control and FMNL1 siRNA for 48 h, and protein lysates were analysed by western blotting and ECL detection using anti-FMNL1 and anti-tubulin antibodies. **c**, Quantification of FMNL1 protein expression relative to tubulin from western blots shown in b. ± SEM, n = 3 samples from 2 independent experiments **d**, U2OS Cherry-HPOS cells were treated with 400 µM oleic acid overnight, saponin permeabilized, fixed and immunostained for FMNL1. Cellular areas containing LDs (R1) or without LDs (R2) are magnified. Scale bar 10 µm and 1 µm (inset). **e**, FMNL1 intensity was quantified in regions with or without LDs and expressed relative to FMNL1 intensities in LD regions. n = 23 cells from 3 independent experiments. **f**, Stable U2OS Cherry-HPOS cells were treated with siControl or siFMNL1 and with 400 µM oleic acid overnight. Cells were permeabilized with

saponin, fixed, immunostained for FMNL1 and imaged by confocal microscopy. Scale bar 5 µm. **g,** Quantification of FMNL1 patches at LDs from images acquired as in (**f**), n ≥50 LDs per siRNA treatment. **h**, Confocal microscopy of stable U2OS Cherry-HPOS cells permeabilized with saponin, fixed and immunostained for myosin light chain and FMNL1. Scale bar 10 µm and 1 µm (inset). Student's t-test, *** $p < 0.001$.

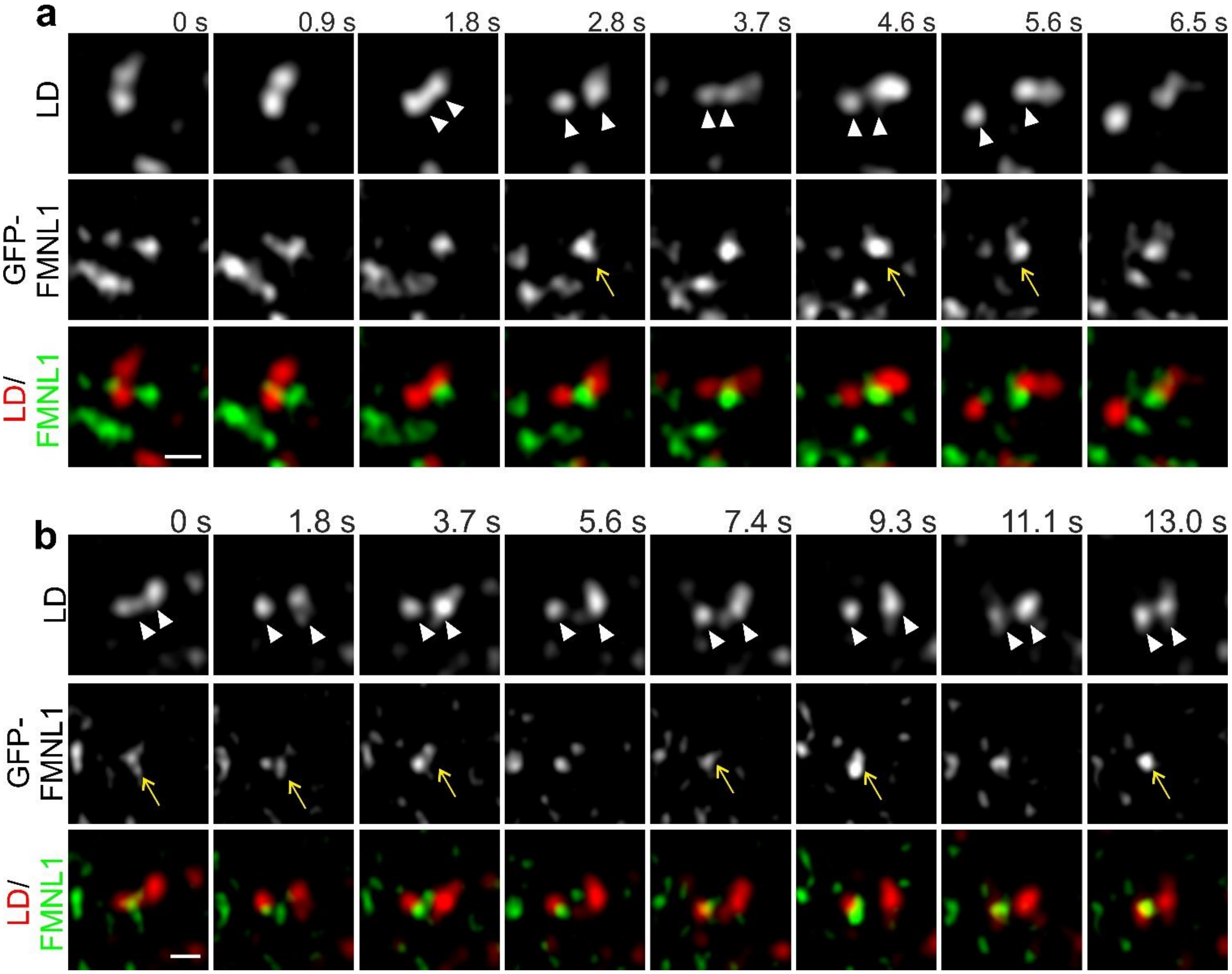

Supplementary Figure 6. FMNL1 dynamics at clustered LDs undergoing dissociation and reassociation. (**a, b**) Live cell Airyscan microscopy of GFP-FMNL1 and LDs in U2OS cells treated with 200 µM oleic acid overnight and images were acquired every 925 ms. Scale bar 0.5 µm. Arrowhead indicates a dissociating LD surrounded by GFP-FMNL1 (arrow). The image sequence displayed in **b** represents the same LD cluster as in **a** at later time points. (See also Supplementary Movies 10, 11).

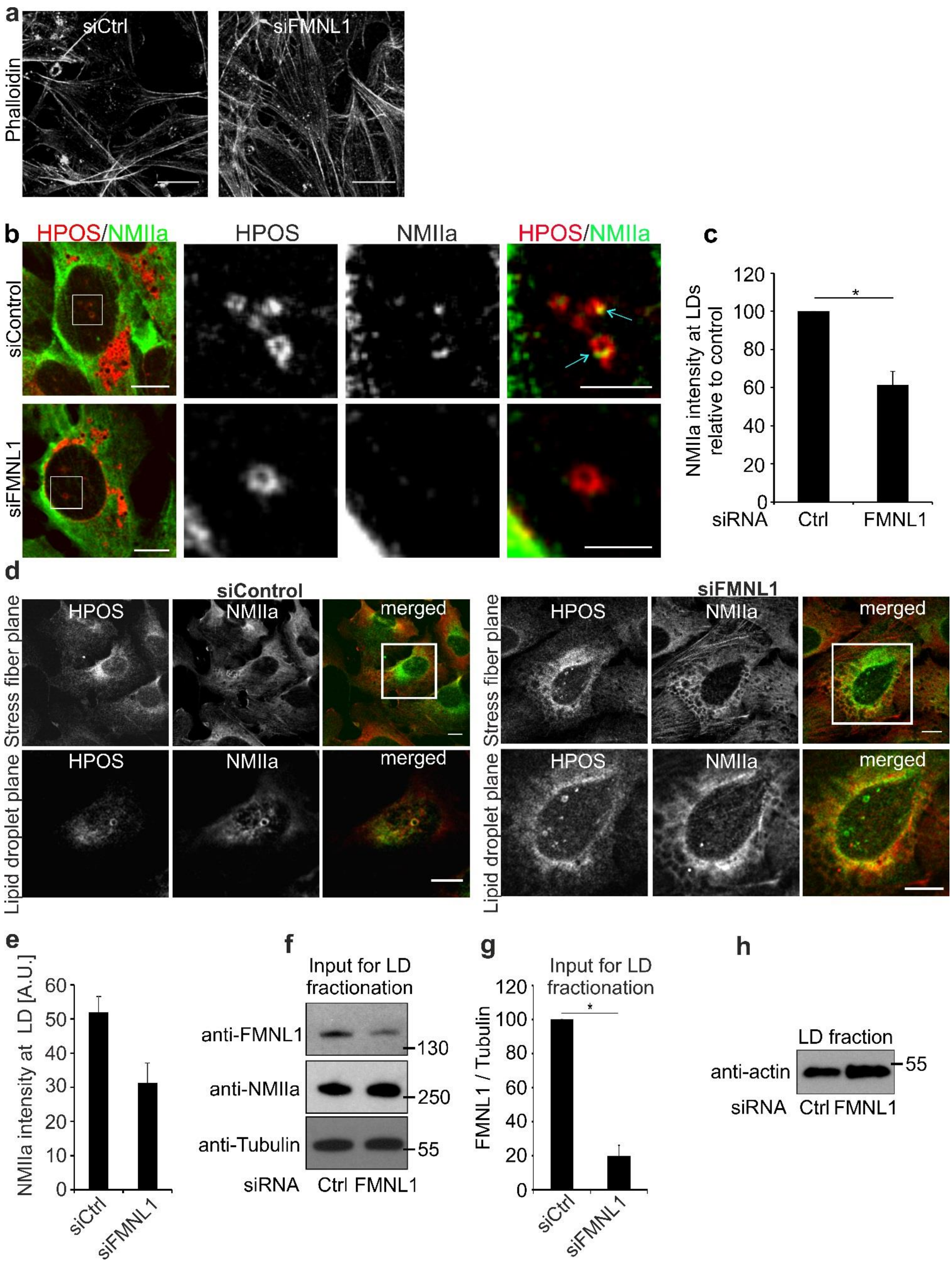
a
siCtrl
siFMNL1
Phalloidin
b
HPOS/NMIIa
HPOS
NMIIa
HPOS/NMIIa
siControl
siFMNL1
c
NMIIa intensity at LDs relative to control
120
100
80
60
40
20
0
*
siRNA
Ctrl
FMNL1
d
siControl
siFMNL1
Stress fiber plane
Lipid droplet plane
HPOS
NMIIa
merged
e
NMIIa intensity at LD [A.U.]
60
50
40
30
20
10
0
siCtrl
siFMNL1
f
Input for LD fractionation
anti-FMNL1
anti-NMIIa
anti-Tubulin
130
250
55
siRNA
Ctrl
FMNL1
g
Input for LD fractionation
FMNL1 / Tubulin
120
100
80
60
40
20
0
*
siCtrl
siFMNL1
h
LD fraction
anti-actin
55
siRNA
Ctrl
FMNL1

Supplementary Figure 7. a, FMNL1 depletion results in more pronounced actin stress fibers as visualized with phalloidin staining, in accordance with previous findings[21]. Stable U2OS Cherry-HPOS cells were imaged using confocal microscopy. Scale bar 20 µm. **b**, Stable U2OS Cherry-HPOS cells were treated with siCtrl or siFMNL1 for 48 h and treated overnight with 400 µM oleic acid. Cells were immunostained for NMIIa. Arrows indicate NMIIa localization at LDs, scale bar 20 µm and 5 µm (inset). **c**, Quantification of NMIIa intensity at LDs above the nucleus from confocal sections as in (**b**). ± S.E.M., n = 3 independent experiments with quantification of at least 85 LDs per treatment. Student's t-test, * $p < 0.05$. **d,** Examples of stress fiber and LD localization of NMIIa upon FMNL1 depletion in stable U2OS Cherry-HPOS cells for 48 h treated with control medium. Scale bar 10 µm and 5 µm (inset). **e,** Quantification of NMIIa intensity at LDs above the nucleus from confocal sections as in (**d**) (n = 17 LDs for both treatments). **f**, Western blot analysis for FMNL1, NMIIa and tubulin used as input for the LD fractionation shown in Fig. 4g . **g**, Quantification of FMNL1 abundance in input samples relative to tubulin. **h**, Western blot analysis of actin in LD fractions prepared as in Fig. 4g. Student's t-test, * $p < 0.05$.

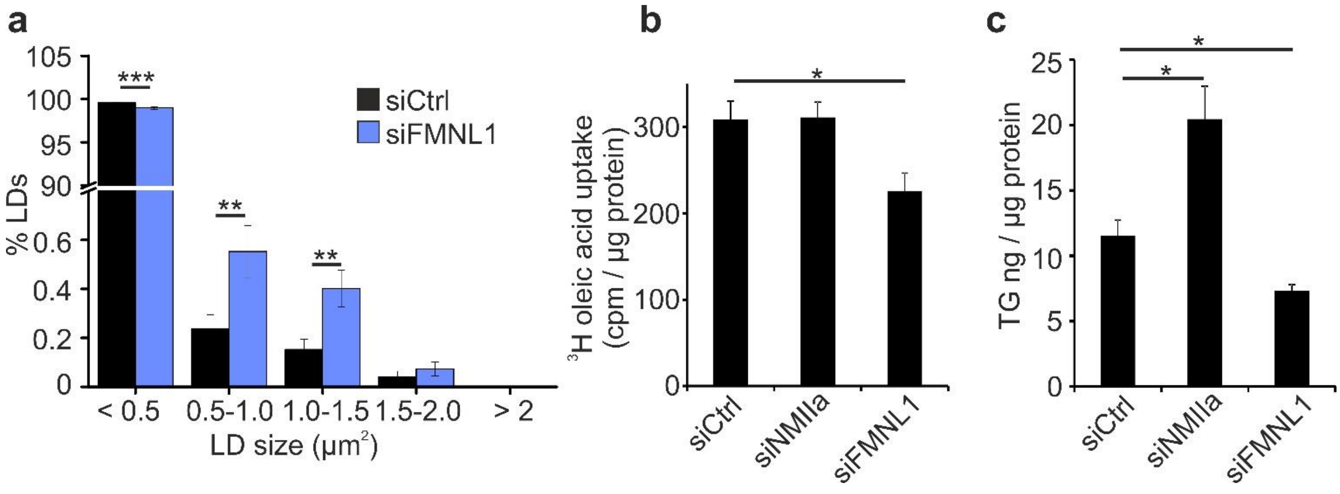

Supplementary Figure 8. a, Automated LD quantification of U2OS cells treated with siCtrl or siFMNL1 for 48 h. ± SEM, n = 329 cells for siCtrl and 314 for siFMNL1 from 3 independent experiments. **b**, ^{3}H-oleic acid uptake in U2OS cells treated with siCtrl, siNMIIa or siFMNL1 for 48 h. ± SEM, n = 8 samples from 2 independent experiments. **c**, Lipid quantification of U2OS cells treated as in b. ± SEM, n = 5 samples from 2 independent experiments. Student's t-test, * $p < 0.05$, ** $p < 0.01$, *** $p < 0.001$

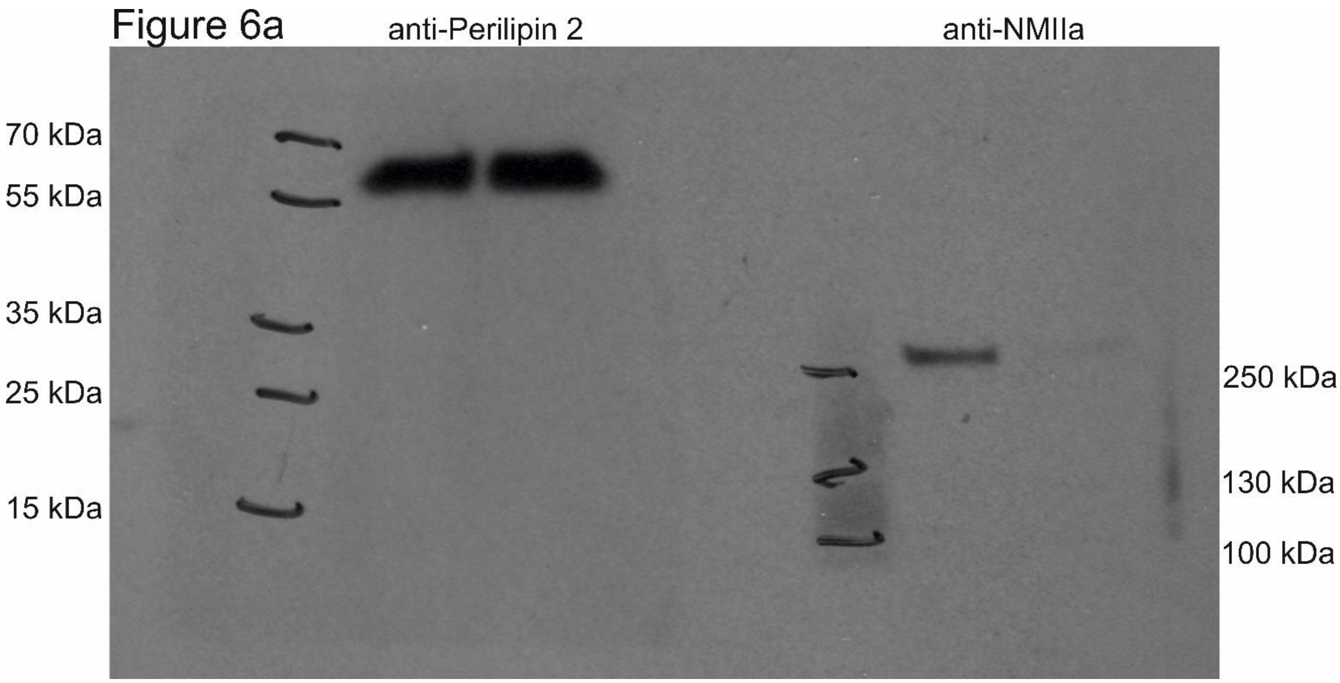

Supplementary Figure 9. Original scan of blots presented in the main figures.

Uniprot:	Name:	Peptides:
P26039	Talin-1	48
P40124	Adenylyl cyclase-associated protein 1	40
P60710	Actin, cytoplasmic 1	33
Q9QXS1	Plectin	33
Q8VDD5	Myosin-9	22
Q9JL26	Formin-like protein 1	21
P68033	Actin, alpha cardiac muscle 1	15
Q61233	Plastin-2	14
P21107	Tropomyosin alpha-3 chain	11
P84096	Rho-related GTP-binding protein RhoG	8
P18760	Cofilin-1 OS=Mus musculus	8
Q60605	Myosin light polypeptide 6	6
P24452	Macrophage-capping protein	6
Q99PT1	Rho GDP-dissociation inhibitor 1	6
O89053	Coronin-1A	4
Q61599	Rho GDP-dissociation inhibitor 2	4
P62962	Profilin-1	4
P60766	Cell division control protein 42 homolog	3
P47753	F-actin-capping protein subunit alpha-1	3
P61161	Actin-related protein 2	2
P59999	Actin-related protein 2/3 complex subunit 4	2
Q3THE2	Myosin regulatory light chain 12B	2
Q62159	Rho-related GTP-binding protein RhoC	2
Q9WV32	Actin-related protein 2/3 complex subunit 1B	2
Q99JY9	Actin-related protein 3	2
P47757	F-actin-capping protein subunit beta	2
P47754	F-actin-capping protein subunit alpha-2	2
P26041	Moesin	2
Q9QY06	Unconventional myosin-IXb	1
Q6ZWR6	Nesprin-1	1

Supplementary Table 1. Actin related proteins associated with LDs. LDs were purified by density gradient centrifugation from lipid loaded Raw 264.7 cell extracts and subjected to mass spectrometric analysis of associated proteins. Uniprot accession numbers, protein names and their peptide counts for actin related proteins detected are shown. NMIIa (Myosin-9) and FMNL1 are highlighted in red.
